# Exploiting Synergy: Immune-Based Combinations in the Treatment of Prostate Cancer

**DOI:** 10.3389/fonc.2014.00351

**Published:** 2014-12-12

**Authors:** Mauricio Burotto, Nishith Singh, Christopher R. Heery, James L. Gulley, Ravi A. Madan

**Affiliations:** ^1^Genitourinary Malignancies Branch, National Cancer Institute, National Institute of Health, Bethesda, MD, USA; ^2^Indiana University Health Arnett, Lafayette, IN, USA

**Keywords:** prostate cancer, immunotherapy, vaccines, radiation, chemotherapy, checkpoint inhibitors, hormonal treatment

## Abstract

Cancer treatment is being revolutionized by the emergence of immunotherapies such as immune checkpoint inhibitors and therapeutic cancer vaccines. Prostate cancer is amenable to such therapeutic approaches. The improved understanding of the relationship between the immune system and tumors has allowed therapeutic targeting of immune checkpoints and tumor associated antigens to be developed. Furthermore, interventions used in prostate cancer are capable of impacting the immune system. As demonstrated by preclinical data and emerging clinical data, radiation therapy, anti-androgen therapy, and chemotherapy can be used with immunotherapies to obtain synergistic results. Current and future clinical trials will further investigate these principles as immunotherapeutics are combined with each other and standard therapies for optimal clinical utility.

## Introduction

Prostate cancer is the most common malignancy in men diagnosed in the United States and is second in cancer related death only surpassed by lung cancer, with 29,480 projected deaths in 2014 (1). Although recent years have seen great advances in treatments for prostate cancer, including second-line chemotherapy, anti-androgen therapies, and radiopharmaceuticals, none of these therapies are curative (2). Nonetheless, there is great potential for these and existing therapies to be used synergistically with immunotherapies already in clinical practice or in late stages of clinical trials. Furthermore, given the lack of significant toxicity seen with therapeutic cancer vaccines and the lack of over-lapping toxicity seen with immune checkpoint inhibitors, it appears possible that immune-based combinations have the potential for improving clinical outcomes without causing patients significant additional side effects. This is very important in a disease such as prostate cancer where symptoms from the disease are generally not present until the late stages (3).

Innate and adaptive immune responses have been studied as a means of prevention and control of tumors (4, 5). The dynamic process of immune activation against cancer can begin with antigen presentation via dendritic cells (DCs) and other antigen presenting cells (APCs) and recognition of those antigens by T cells via the T cell receptor (TCR) (6–8). A subsequent maturation signal [including toll like receptors (TLRs) or endogenous factors such as high mobility group (HMG) proteins or adenosine tri-phosphate (ATP) from the dying cells] is required from APCs to induce adequate activation of T cells (6). Peptide antigen bound to the major histocompatibility complex (MHC)-derived molecule is presented to the TCR. This recognition and interaction is the central event that leads to effector cellular immunity (7). In addition, the interaction and activation of the B7 family of co-receptors is crucial to initiate sufficient T cell activation, and in the case of tumors, an anti-tumor response (8). The process of T cell mediated tumor elimination may also involve Fas mediated apoptosis, release of cytotoxic molecules such as perforins, and indirect cellular killing through release of interferon-gamma (7). There are limitations in immune mediated control of tumors, such as tumor mediated immune suppression (6). Therefore, immune strategies including combination therapies with cytoreductive agents can be employed to treat cancer have to help cytoreduce the tumor and overcome some of these immune suppressive obstacles.

Prostate cancer is an excellent tumor target for immune-based therapies. First and foremost, prostate cancer has an indolent disease course, which allows the immune system to generate an immune response. In addition, prostate specific antigen (PSA) allows for detection of disease when the cancer is at the micro-metastatic level, allowing for small volumes of disease to be treated. Given that increasing levels of tumor burden carry increasingly immune suppressive attributes, starting immune-based treatments with minimal tumor may be advantageous (9). Also, prostate cancer has well characterized tumor associated antigens (TAAs), which can serve as therapeutic immunologic targets (10–12). Together these characteristics may explain some of the preliminary clinical success with immunotherapy in prostate cancer, but more importantly they may be indications of true therapeutic potential when optimized as part of combination regimens.

## Immunotherapy in Prostate Cancer

### Therapeutic cancer vaccines

The promise of immune-based therapies to control cancer has come to fruition in recent years with the regulatory approval of sipuleucel-T in prostate cancer and immune checkpoint inhibitors in melanoma (13, 14). Therapeutic cancer vaccines such as sipuleucel-T are designed to enhance immune recognition of specific TAAs, leading to immune-mediated killing of cancer cells. Sipuleucel-T is a therapeutic cancer vaccine generated from a patient’s own immune cells, which are collected via apheresis, activated in *ex vivo* fashion, and then re-infused into the patient. The *ex vivo* processing of immune cells is designed to enhance immune recognition of the TAA prostatic acid phosphatase (PAP) (11). A full course of therapy with sipuleucel-T consists of three infusions every other week for 1 month. Early clinical trials demonstrated the safety and tolerability of this treatment, which had minimal side effects (15). Although two initial phase III trials in metastatic castration-resistant prostate cancer (mCRPC) did not meet their primary endpoint of improved time to progression, there was a suggestion that they improved survival (15). In a third clinical trial, sipuleucel-T extended the lives of patients with mCRPC relative to placebo (25.8 vs. 21.7 months; HR = 0.78; *p* = 0.03) leading to FDA approval in patients with minimally symptomatic or asymptomatic mCRPC (13).

Another vaccine in late stages of clinical development is PROSTVAC (PSA-TRICOM), a pox-viral vaccine developed to stimulate the immune system via *in vivo* immunologic stimulation at that is designed to enhance targeting of PSA (10). Unlike Sipuleucel-T, PROSTVAC consists of recombinant pox viruses that are injected into a patient subcutaneously and does not require *ex vivo* processing of immune cells (16). Once within the patient, the pox viruses infect including immune cells. Subsequently, recombinant genetic material within the virus is translated within the cytoplasm of the DCs (10). This genetic material encodes for PSA, the immunologic target, and 3 T cell co-stimulatory molecules that have demonstrated the ability to enhance T cell activity in a synergistic manner (17). The end result is that the infected DC serves as an APC, displaying PSA in an immunologic context, along with co-stimulatory molecules, leading to TAA-specific immune activation and targeted T cell destruction of tumor cells. Early clinical trials demonstrated that common side effects were limited to self-limiting injection site reactions and flu-like symptoms (18). Subsequent phase II studies demonstrated the ability of this vaccine to improve T cell recognition of PSA (19). In addition a randomized, multi-center trial (*n* = 125) was conducted in chemotherapy-naïve mCRPC patients with PROSTVAC vs. placebo. The results were favorable, suggesting an improvement in overall survival (25.1 vs. 16.6 months; HR = 0.56, *p* = 0.0061) (20). Together these two studies have led to a randomized phase III trial in chemotherapy-naïve mCRPC (NCT01322490).

### Immune checkpoint inhibitors

Another immunotherapeutic approach involves the disruption of immune regulation via monoclonal antibodies design to inhibit immune checkpoints. Such therapies do not provide the immune system with an immunologic target such as a TAA, but rather allow amplified or sustained T cell activation in a non-specific fashion. Although this can lead to a more aggressive response, it may lead to immune related adverse events (irAEs) such as rash, colitis, and endocrinopathies. Ipilimumab is the first-in-class agent designed to bind to CTLA-4, a molecule expressed by T cells after activation. CTLA-4 binds CD80, a co-stimulatory molecule present on APCs, and prevents CD80 from binding CD28, a co-stimulatory molecule present on T cells. CTLA-4 is a competitive inhibitor of the activating signal and results in T cell suppression or inactivation (21). Ipilimumab binding to CTLA-4 prevents the natural auto-regulatory interactions of T cells with APCs. This blockade of CTLA-4 increases the likelihood of greater, albeit non-specific, T cell activity (21). Such activity has potentially profound effects. Indeed, CTLA-4 knock-out mice ultimately die from lymphocytic infiltration of their organs (22).

Ipilimumab was FDA-approved for the treatment of metastatic melanoma based on an overall survival benefit compared to a peptide-based vaccine (GP100), which served as an active control, although there was no change in short term disease progression. Immune-related adverse events (irAEs) are the toxicities uniquely seen with the use of checkpoint inhibitors. These events are mechanism-based and chiefly include skin and mucosal rash (47–68%), diarrhea/colitis (all grades, 44%), hepatotoxicity (3–9%), and hypophysitis (1–6%) (14). Immunosuppressive agents such as systemic steroids are the standard treatment for many irAEs, which are thus reversible (23). For irAEs that impact endocrine organs, replacement hormones may be required.

Ipilimumab has also been evaluated in prostate cancer. Preliminary trials suggested activity as well as common irAEs seen in studies in other tumor types (24–27). A recently completed phase III trial evaluated ipilimumab combined with low dose radiation as a potential immune enhancer (28). The placebo controlled study was conducted in late stage mCRPC patients who had already been treated with docetaxel. While the study was negative, there was a trend to improved survival (11.2 vs. 10 months for placebo; HR = 0.85; *p* = 0.053), which was the primary endpoint of the study. A *post hoc* subgroup analysis suggested that patients with indolent disease features who received ipilimumab had a substantial improvement in survival compared to those who received placebo. These data are encouraging because an on-going study is evaluating ipilimumab in an earlier stage of disease, chemotherapy-naïve mCRPC, has completed accrual and results are pending (NCT01057810).

Agents blocking programed cell death protein-1 (PD-1) and its ligand PD-ligand-1 (PD-L1) are other examples of immune checkpoint molecules shown to have anti-tumor activity in solid malignancies and appear to have an improved safety profile compared to anti-CTLA-4 (29, 30). Lymphocyte activation gene 3 (LAG3) and B7-H3 are also novel targetable immune checkpoint molecules currently in clinical development, while B7-H4 and T cell membrane protein 3 (TIM3) inhibitors are in preclinical development (31, 32). These treatments may also be investigated in the future in prostate cancer.

## Biological Basis and Rationale of the Combinatorial Immunotherapy Approach

**Figure 1:**
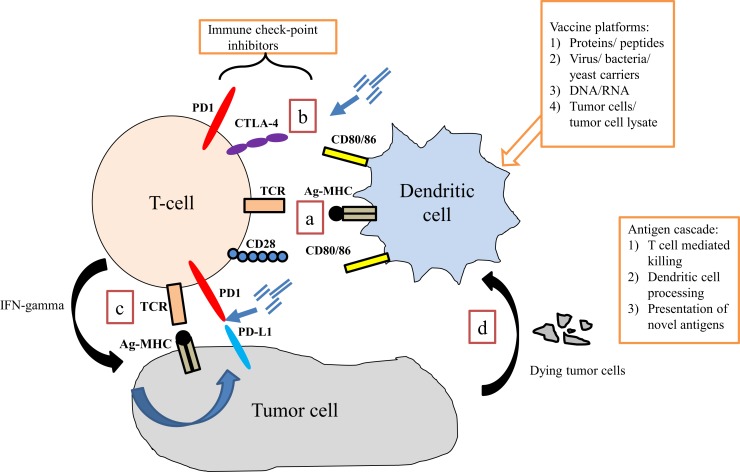
**Interaction of immune cells with tumor cells**. (a) Dendritic cells present antigen via MHC class II to T cells via the T cell receptor (TCR). Binding of co-stimulatory signaling molecules (signal 2) is required for adequate T cell activation to occur. (b) Immune checkpoints are present at several points of the immune response, which regulate T cell activity. Blocking these interactions may enhance anti-tumor T cell activity. (c) In response to activation via dendritic (or other antigen presenting) cell signaling, T cells up-regulate specific TCRs and produce interferon-gamma (IFN-γ), which induces tumor cells to express programed death receptor 1 ligand (PD-L1), perhaps making it a therapeutic target. (d) Tumor cell destruction results in debris that is engulfed by APCs (including dendritic cells), processed, and presented back to T cells. This process is the basis for an immunotherapy-induced process known as antigen-cascade/antigen spreading.

The opportunity to combine more standard, non-immunologic therapies with immune-based therapies is derived from growing understanding that these standard therapies may enhance immune activity. Some cytotoxic therapies may induce an immunologic form of cell death that serves to feed an on-going immune response (33). Other therapies may kill cancer cells in a way that leads to the release of “molecular danger” (34) signals that may improve immune anti-tumor activity (35). Other therapies may impact the tumor microenvironment in ways that enhance immune recognition or killing of tumor cells (35, 36). These characteristics have been extensively studied in the context of the standard treatments used in prostate cancer and help provide a strong rationale for immunologic combinations to treat this disease (Figure 1; Table 1).

**Table 1:** **Effects on immune system of different interventions in cancer**.

Intervention	Immunologic impact	Example of intervention
Hormone manipulation	Increased thymic T cell production (38)	LHRH antagonist
	Potentiation of T cell response (39)	
	Decreased in Tregs [CD4+CD25+] (41)	
Chemotherapy	Increase antigen expression (77)	Cyclophosphamide Docetaxel Oxaliplatin
	APC activation (75)	
	Decrease in Tregs [CD4+CD25+] (71)	
	Induce ICD (75)	
Radiation therapy	DC activation (56)	External beam radiation IMRT
	Release of danger signal (HMGB1) (58)	
	Increase calreticulin expression (75)	
Immune- checkpoint blockade	Increased aggressiveness immune response (86)	Ipilimumab Anti-PD-1 Anti-PD-L1
	Decreased Inhibitory signal of T effector cells (84)	

*LHRH, luteinizing hormone-releasing hormone; IMRT, intensity-modulated radiation therapy*.

## Anti-Androgen Therapy

Androgens fuel the proliferative mechanisms of prostate cancer and thus anti-androgen therapy is the cornerstone of prostate cancer therapy (37). A number of clinical and preclinical studies have demonstrated the potential synergy between anti-androgen therapy and immune stimulating therapies. These findings support combining immune-based treatments with several standard therapies in prostate cancer.

Recent preclinical studies have confirmed the earlier findings and shed light on the cellular changes that underlie the effect of anti-androgens. Following surgical castration in aged mice, reversal of thymic atrophy, restoration of thymic architecture, normalization of thymocyte differentiation, proliferation, and levels of apoptosis, are observed (38). Increased thymic import and restoration of peripheral thymocyte pool and function have also been noted. Similarly, LHRH treated older men, at 4 months showed a significant increase in total peripheral lymphocytes (*p* ≤ 0.05), T cells (*p* ≤ 0.01) (predominantly naive CD4+, as well as both naive and memory CD8+ T cells), and natural killer (NK) cells (*p* ≤ 0.05) (39). In some patients, the changes are associated with a 25% increase in naïve T cells as measured by TCR excision circles, a byproduct of new T cell production by the thymus.

Androgen ablation in mice mitigates tolerance of the tumor to the immune system, suggesting that immunotherapy might be more efficacious if used in combination with hormone ablation (40). In mouse models of endogenous prostate cancer, accumulation of functional T cells in the prostate glands is seen at about 2.5 weeks after castration. It is hypothesized that the antigens released by dying prostate cancer cells may stimulate the CD8 T cells (41). A functionally effective, intra-prostatic population of T cells can be augmented by *in situ* injection of vaccine in PTEN-knock-out mouse models (42).

Recently, modern anti-androgens such as enzalutamide have been developed and have demonstrated the ability to prolong life in mCRPC with minimal side effects (43, 44). While these advances have revolutionized the treatment landscape for prostate cancer it has also created opportunities to explore synergy with immunotherapy. Studies in murine models have demonstrated that enzalutamide, like androgen deprivation therapy (ADT), enhances the production of naïve T cells from the thymus (45, 46). Clinical trials combining therapeutic cancer vaccines and enzalutamide are currently underway (NCT01875250 and NCT01867333).

In the human studies, ADT has demonstrated an immune response in the form of a mononuclear cell infiltrate, a response that peaks at 2–3 weeks (47). ADT modulates the subtype of immune cells that home on prostate as shown in a computer-assisted analysis of prostatectomy specimens in 75 patients (ADT-treated, 35; control, 40) (48). Compared to the controls, ADT-treated patients had a significant increase in relative density of CD3(+) (*p* < 0.001) and CD8(+) T-lymphocytes (*p* < 0.001) as well as CD68(+) macrophages (*p* < 0.001). A prognostic correlation was sought as well in the study. Elevated numbers of CD56(+) NK cells were found to be associated with a lower risk of prostate cancer progression (*p* = 0.044), while a high density of CD68(+) macrophages was related to an increased risk of biochemical recurrence (*p* = 0.011).

The safety and efficacy of autologous cellular cancer immunotherapy (sipuleucel-T) has been studied in combination with ADT in patients with non-metastatic castration-sensitive prostate cancer (nmCSPC). A double-blind, controlled, multi-center study randomized 176 patients with biochemical recurrence (rising PSA) after prostatectomy into sipuleucel-T and placebo arms. The treatments were instituted after a 3–4-month run-in period of androgen suppression therapy (49). Compared to the placebo arm, subjects who were given sipuleucel-T had a 48% increase in PSA doubling time following testosterone recovery (155 with sipuleucel-T vs. 105 days, *p* = 0.038). These data suggest that sipuleucel-T can slow the growth of cancer over time and this hypothesis is corroborated by additional data from clinical studies with PROSTVAC (50).

At least two phase II randomized clinical trials are evaluating the impact of sequencing ADT on systemic immune responses. The first study (NCT01431391) is a phase II randomized trial that will attempt to determine if ADT before or after vaccination with sipuleucel-T leads to better immune responses in nmCSPC patients. The results of this study could have implications for future immunotherapy studies involving initiation of ADT. The second study (NCT01487863), also a randomized phase II investigation, will evaluate the use of concurrent or sequential abiraterone and prednisone with sipuleucel-T in mCRPC. Preliminary data suggested that abiraterone and prednisone when given concurrently with sipuleucel-T did not diminish immune stimulation seen with sipuleucel-T alone (51). These data are important because they start to address a prevailing dogma, which suggests that prednisone at 10 mg daily (an important part of several mCRPC regimens) may diminish immune responses induced by sipuleucel-T, although there are no clinical data to support these concerns.

Combined androgen blockade involves the concomitant administration of ADT and an androgen receptor antagonist (ARA), such as nilutamide. One clinical trial combined androgen blockade (CAB) with vaccines, using cross-over design (52). In the trial, the patients who started on nilutamide first with vaccine added only at PSA progression had a median time to treatment failure of 5.2 months. In contrast, the median time to treatment failure (defined by rising PSA or development of a metastatic lesion) with the combined therapy was 13.9 months in the vaccine arm when nilutamide was added at PSA progression thus favoring a strategy of early introduction of vaccine. A follow-up analysis revealed a 75% 5-year survival rate for patients treated first with vaccine in comparison to 43% 5-year survival rate for patients who received nilutamide first and had vaccine added at a later time (53). These data suggest that synergy of anti-androgen therapy and immunotherapy may be optimized when the combinations are deployed earlier in the disease course. As previously discussed, newer ARAs such as enzalutamide have been developed and combinatorial trials with these newer agents are on-going (NCT01875250 and NCT01867333).

## Radiation Therapy

Radiation therapy kills cells by inducing lethal DNA damage, which in turn leads to apoptosis and cell death (54). Additional effects include inflammation in the microenvironment, sub-lethal injury to some of the tumor cells, changes in the tumor vasculature, and potentially activation of immune cells (55, 56). Radiation can up-regulate TAAs, co-stimulatory molecules, Fas, MHC moieties, cytokines, chemokines, and adhesion molecules, and down-regulate regulatory T cells (Tregs), all of which renders tumors susceptible to immune attack. The release of TAA and recruitment of APCs at the site of the debris, necrosis and inflammation (increased expression of IL-1β and TNF-α) at the site of radiation, can directly activate the immune system and cause immune cell induced cytolysis (57). Specifically, the up-regulation of immunomodulatory surface molecules such as MHC type I and death receptors such as Fas have been demonstrated to mediate specific immune mediated killing (58). Garnett et al. showed that sub-lethal doses of radiation change the phenotype of the human tumor cell lines increasing, mucin-1, CEA, and MHC class I. These changes augment the killing of the tumor cell by specific CD8(+) T cells restricted or specific to this phenotype (59).

The combinatorial efficacy of radiation and adoptive cell transfer of cytotoxic T cells in a preclinical model was demonstrated by Chakraborty et al. (60). Sub-lethal doses of irradiation in a mouse adenocarcinoma model increased the anti-tumor activity of ACT Ag-specific cytotoxic T cells by utilizing the Fas/Fas ligand pathway of tumor death. Radiation and immunotherapy using *Listeria monocytogenes*-based vaccine (ADXS31-142) was investigated in a mouse model of prostate cancer. Together, they demonstrated a greater delay in tumor growth, increase in specific cytotoxic T cells and high level of interferon production compared to the animals that did not receive the combined treatment (61).

Gulley et al. tested the combination of definitive external beam radiation therapy plus vaccination in localized prostate cancer patients (62). In this randomized phase II trial patients received radiation alone or radiation combined with a viral vaccine construction (PSA gene inserted and B7.1 co-stimulatory gene). The results showed an increased PSA-specific T cell response in the combination compared with the radiation alone arm (*p* < 0.0005). In addition to the demonstration of immunologic responses, the combination was safe without major toxicity; the only grade 3 events were attributed to the IL-2 given as adjuvant to the vaccine. A similar experience by Lechleider et al., using a lower dose of IL-2 during several days (metronomic) with radiation therapy in patients with localized prostate cancer showed significant increases in PSA-specific-T cell responses and lower toxicities (63).

In addition to external beam radiation, therapeutic radiation can also be delivered via radiopharmaceuticals. Agents such as samarium-153 EDTMP are designed to localize to remodeled bone, which are likely sites of metastatic cancer. Once there, the radiation component (samarium-153) emits radioactive particles that can kill cancer cells via DNA damage. This agent is approved for palliation in patients with mCRPC (64). Like external beam radiation, radiopharmaceuticals have been shown to also alter the phenotype of cancer cells and enhance killing by immune cells (65). A clinical trial has combined PROSTVAC with samarium-153 in patients with mCRPC who had progressive disease on standard front-line chemotherapy, docetaxel. Preliminary data suggest that samarium-153 combined with vaccine prolonged progression free survival (PFS) more than samarium-153 alone (3.7 vs. 1.7 months; HR = 0.48, *p* = 0.034) (66). These data are especially interesting given recent the innovation of radium-223, which has similar properties as samarium-153, but a more reasonable side effect profile. Furthermore, this agent has demonstrated an ability to improve survival in mCRPC, raising the intriguing possibility of future combination studies involving radium-223 and immunotherapy (67).

A completed phase III trial in mCRPC has already used radiation as a form of immune adjuvant to potentially enhance the clinical impact of immunotherapy (28). This trial was also conducted in men with mCRPC who had previously progressed on front-line chemotherapy. Patients were randomized to external beam radiation to isolated bone lesions with either ipilimumab or placebo. The role of radiation was not palliative, but the 8 gy dose was designed to alter the cancer cells within the lesions, as previously discussed, with the goal of potentiating an immune response (68). The trial did not meet its primary endpoint, despite a trend toward an overall survival benefit in patients treated with radiation and ipilimumab compared to radiation and placebo (11.2 months vs. 10 months; *p* = 0.053, HR = 0.85) (28). There was also a trend to improved survival seen in patients with more indolent disease features suggesting that future studies may be optimized by focusing on similar populations. Although the results fell short of expectations, this study provides hypothesis-generating data for future studies and evidence that using radiation as an immune adjuvant is feasible in large randomized trials.

## Chemotherapy

Cytotoxic chemotherapies have been traditionally considered to be immunosuppressive, but emerging data are now starting to dispel that dogma as well. Many chemotherapeutics agents like cyclophosphamide and methotrexate cause leukopenia and lymphopenia and have been used for many years as immunosuppressive agents (69). Others such as the nucleoside analogous cladribine and fludarabine cause more profound T cell depletion (70). In addition to the known effects on the immune system, accumulating evidence demonstrates that cytotoxic drugs may augment the anti-cancer immune response and potentially be synergistic with immunotherapy approaches (33).

Notably, with cytotoxic therapy, the decrease in the host’s lymphocyte population can be selective, as has been shown in case of cyclophosphamide. Specifically, the drug diminishes the T cell subpopulation that is CD4+ CD25+ FOXP3+ T cells, also known as Tregs (71). Tregs suppress the activation of the immune system and prevent pathological self-reactivity; a good example of this paradigm is in the prevention of autoimmune diseases. Initial observation as to the selective cytotoxicity of cyclophosphamide on Tregs was described in a seminal paper published by North et al. (72). Using a mouse model, these data demonstrated that the combination of adoptive cell transfer of T cells in the presence of cyclophosphamide was better in achieving tumor regression compared to the control. Several strategies in modern immunotherapy against cancer involve some type of decrease in the number and function of Tregs with cyclophosphamide (73, 74).

Another key concept in the combination of immunotherapy and chemotherapy is immunogenic cell death (ICD), which refers to the capacity of tumors cell to elicit an immune stimulatory response after death or lethal injured induce by cytotoxic therapies. Based on several hypothesizes, critical steps in ICD may involve exposure of calreticulin at the surface of dying tumors cells, HMGB1 secretion, and ATP release, all of which increase the uptake of tumor antigens by the surrounding DCs and initiate an immune response (75, 76). The *in situ* immunogenic effects observed with agents such as oxaliplatin, mitoxantrone, cyclophosphamide, and doxorubicin as well as certain types of radiation on the susceptible host cells have been explained via the induction of ICD (75, 76).

Cytotoxic drugs may also augment anti-tumor immune response via the phenomenon of immunogenic modulation. As opposed to lethality-associated ICD, immunogenic modulation results from the sub-lethal effects of chemotherapy on the tumor that brings about a change in the surface antigen expression (phenotypic change). Docetaxel, which is one of the two cytotoxic drugs approved in metastatic prostate cancer, has been demonstrated to replicate this process in low doses in preclinical models through up-regulation of cell surface molecules like ICAM-1, MUC-1, and MHC class 1 molecules (77). Hodge et al. demonstrated the effects of docetaxel administration in non-lethal doses in a variety of cell lines and xenograft models. Tumor cells did not necessarily die by ICD, but rather there was an increased sensitivity to antigen-specific cytotoxic T cell killing and a broadening of the immune response resulting in the targeting of multiple antigens (antigen-cascade/spreading discussed later) (78).

Clinical experience in prostate cancer utilizing the combination of chemotherapy and vaccines has been reported by Arlen et al. The phase II trial randomized 28 patients to docetaxel plus viral-vector based vaccine (vaccinia and fowlpox virus expressing PSA gene and the co-stimulatory gene B7.1) vs. vaccine alone. The combination was deemed safe; immune responses, as measured by antigen-specific-T cell activation against PSA antigen were equivalent in both arms with a median PFS in the docetaxel arm of 6.1 months, which was favorable when compared with a historic control (3.7 months) (79). Interesting data from a study involving breast cancer may have particular relevance in prostate cancer due to the use of docetaxel chemotherapy in combination with PANVAC, a pox-viral vaccine, targeting MUC-1 and CEA and encoding three co-stimulatory molecules, similar to PROSTVAC, and suggest the benefit of the combination in metastatic breast cancer (PFS of 6.6 vs. 3.8 months, HR = 0.67) (80, 81).

The potential for immunotherapy combinations with chemotherapy in prostate cancer may have more potential in light of recent data that suggest docetaxel in newly metastatic castration-sensitive disease (with high volume tumor) have substantially delayed disease progression (49.2 vs. 32.2 months; HR = 0.60) (82). Given that some data suggest that immunotherapy may have its optimal impact early in the disease process, there may be natural opportunities for combination studies in this disease setting in the future (53, 83).

## Combining Immunotherapies: Vaccines Plus Checkpoint Blockade

The rationale for the combination of vaccines and checkpoint inhibition is based on the complimentary mechanisms of these therapies. Vaccines are intended to activate immune cells and ipilimumab is designed to increase T cell activation and killing by blocking immune auto-regulatory mechanism through CTLA-4 blockade (84). Preclinical evidence attests to the ability of anti-CTLA-4 antibodies in improving cytotoxic T-lymphocytes (CTLs) avidity as well. Since high avidity CTLs may have more pronounced anti-tumor efficacy, it provides additional justification to combine ipilimumab and vaccines in clinics as a therapeutic maneuver against prostate cancer (85–87).

The rationale of combining therapeutic cancer vaccines with ipilimumab (to augment T cell avidity) was tested in a Phase I study that combined GVAX with escalating doses of ipilimumab (0.3–5 mg/kg) in patients with mCRPC (27). Seven of 28 patients (25%) who received either 3 or 5 mg/kg ipilimumab had PSA declines of ≥50% while 2 patients showed a clear regression of bone metastases. irAEs were similar in incidence and character as had been previously observed with single agent ipilimumab.

In a second trial involving patients with mCRPC, PROSTVAC was evaluated in combination with ipilimumab in escalating doses of 1–10 mg/kg (25). No dose-limiting toxicities were seen and again side effects typical of ipilimumab were observed. Fourteen (58%) of 24 chemotherapy-naive patients had PSA declines out of which 6 (25%) had declines >50% and 2 of these 6 had declines >90%. In addition, the median survival in these patients with mCRPC was >34 months, which compares favorably to single agent immunotherapy trials in the same population (19, 20, 25).

Together, these trials suggest that, when used in combination, therapeutic cancer vaccines do not enhance the toxicity of anti-CTLA-4 antibodies. Preliminary evidence of efficacy for this combination strategy of immune-checkpoint inhibition and vaccine needs to be confirmed in large randomized studies. In addition, anti-PD-1 and anti-PD-L1 are also in clinical development, with the first agent having been just approved by the FDA in melanoma (29, 30, 88). Given that these agents have demonstrated less toxicity, future combinations with vaccines and anti-PD(L)1 are likely to be clinically evaluated (29, 30, 88).

## Antigen-Cascade/Spreading

Perhaps the least appreciated aspect of immunotherapy is its potential to increase its therapeutic breadth over time, which is a significant distinction from other therapeutics. This can be accomplished by additional antigens being incorporated into an immune response as cancer cells are killed by immune cells or other forms of cytotoxic therapy. As the active immune response encounters new antigens in the tumor microenvironment, they could be included as new immunologic targets for the next generation of immune cells (89). In this manner, immune-based therapies can be more dynamic than cytotoxic therapies, which often have a limited range of therapeutic targets that can ultimately be circumnavigated by the intrinsic heterogeneity likely present within all tumors (90, 91). Although antigen-cascade has been seen in several trials, in multiple cancers, and associations have been made with positive outcomes, additional prospective data are required to determine its potential role as a biomarker or intermediate marker of response (62, 92–94). At this point, observations of antigen-cascade provide proof of concept of how immunotherapy can be used to induce a biologically diverse anti-tumor effect. Future trials, perhaps involving combinations, will look to build on this immune response.

## Conclusion

Immunotherapy in prostate cancer has emerged as a viable therapeutic option and with multiple agents in the late stages of clinical development it is possible the immunotherapeutic portfolio for this disease could expand in the near future. The potential to develop immune-based synergistic combinations in prostate cancer could further optimize the clinical development of these immune enhancing therapies. At each stage of prostate cancer there is sound preclinical rationale for developing such combinations, including newly diagnosed patients (radiation), nmCSPC (ADT), and mCRPC (anti-androgen therapy and docetaxel) (Table 2). The future role of immunotherapy may be in improving the efficacy of these standard therapies. But perhaps the greatest potential for these combinations will be their use in patients with localized disease to perhaps increase the cure rate of the disease or induce functional cures allowing men to live longer lives with prostate cancer. In the best case scenario, immune stimulating therapies can help turn immune cells into sentinels who guard against the cancer inducing morbidity and mortality. Future randomized trials with immune-based combinations will help determine if such regimens can truly revolutionize how prostate cancer is treated and perhaps more importantly, how often localized disease can be cured.

**Table 2:** **Clinical trials using immune combinations in prostate cancer**.

Author	Study type population (Number of patients)	Interventions	Outcomes and comments
**RADIATION COMBINED WITH IMMUNOTHERAPY**			
Gulley (62)	Phase II RCT localized disease suitable for radiation [30]	Definitive EBRT ± vaccine	Threefold increase in PSA T cells vs. no detectable increase in EBRT arm (*p* < 0.0005)
Lechleider (63)	Phase II localized disease suitable for radiation [30]	Definitive EBRT vaccine IL-2 (metronomic low dose)	Safe administration and induction of PSA-specific T cells
			Demonstration of reactive T cells against XAGE-1 and PAGE-4
Heery (66)	Phase II RCT mCRPC (bone disease) [68]	Sm-153 ± vaccine	Increased PFS with Sm-153 + vaccine compared to Sm-153 alone
Kwon (28)	Phase III RCT in mCRPC after docetaxel [799]	Low dose radiation ± ipilimumab	Negative phase III trial, however a trend to OS (HR 0.85 *p* = 0.053)
			Benefit in subpopulation of patients with favorable prognosis
**ANDROGEN DEPRIVATION THERAPY COMBINED WITH IMMUNOTHERAPY**			
Mercader (47)	Phase II T1–2b without previous treatment [35]	ADT prior to RP	Increase T cell and mononuclear cell infiltrates plus tumor atrophy and involution
Madan (53)	Phase II RCT nmCRPC [42]	Vaccine vs. nilutamide (with cross-over)	Improved survival in patients receiving vaccine earlier in the disease course
Beer (49)	Phase II RCT CSPC with PSA recurrence only [176]	ADT ± sipuleucel	Data suggest that vaccine slows the growth rate of tumors (prolonged PSA doubling time)
Small (51)	Phase II RCT asymptomatic/minimally symptomatic mCRPC [63]	Sipuleucel T + AAP (concurrent) Sipuleucel T → AAP (sequencial)	Preliminary data suggest that 5 mg prednisone BID did not diminish APC activation or CD54 up-regulation
**CHEMOTHERAPY COMBINED WITH IMMUNOTHERAPY**			
Arlen (79)	Phase II mCRPC in progression [28]	Docetaxel + vaccine vs. vaccine	No decrease in generation of antigen-specific T cells when docetaxel was added to vaccine

*RCT, randomized clinical trial; EBRT, external beam radiation therapy; mCRPC, castration-resistant prostate cancer; PFS, progression free survival; OS, overall survival; Sm-153, samarium-153; HR, hazard ratio; ADT, androgen deprivation therapy; RP, radical prostatectomy; CSPC, castration-sensitive prostate cancer*.

## Conflict of Interest Statement

The authors declare that the research was conducted in the absence of any commercial or financial relationships that could be construed as a potential conflict of interest.
